# Environmental DNA (eDNA) dataset of foraminiferal diversity and distribution from the mining-impacted estuaries of Goa, west coast of India

**DOI:** 10.1016/j.dib.2024.110541

**Published:** 2024-05-20

**Authors:** Tushar Kaushik, Vaishnavi Dixit

**Affiliations:** a Biodiversity and Palaeobiology Group, Agharkar Research Institute, Pune 411004, Maharashtra, India; b Savitribai Phule Pune University, Pune 411007, Maharashtra, India

**Keywords:** Metabarcoding, Environmental DNA, High-throughput sequencing, Illumina platform, Tropical, Coastal India

## Abstract

The foraminiferal environmental DNA (eDNA) metabarcoding based on high-throughput sequencing (HTS) is a powerful tool to unravel the hidden genetic diversity and environmental lineages. Results from the eDNA approach provided valuable insight into an unplumbed diversity of soft-bodied monothalamous foraminifers [1]. Micropaleontologists overlooked monothalamids due to their soft organic and/or finely agglutinated test, which often gets destroyed during routine morphological investigations [2]. On the other hand, some foraminifera taxonomists or studies included monothalamids (soft-shelled species) in ecological and diversity investigations ranging from deep-sea locations to coastal marine habitats [1], [3], [4]. Here, we document our metabarcoding analysis of foraminiferal diversity and abundance from the mining-affected estuaries of the Indian state of Goa. High-throughput sequencing using the Illumina platform indicated the overwhelming abundance of monothalamous foraminifers in the studied estuarine sediments. For the first time, such detailed data of the foraminiferal diversity utilizing sedimentary environmental DNA (eDNA) methods was carried out in India. The raw sequence data used for analysis is available in NCBI under the Sequence Read Archive (SRA) with the BioProjects and SRA accession number: PRJNA1040471. The presented data may be used as baseline information for eDNA-based biomonitoring and biodiversity assessment surveys from Indian marine habitats across time and space.

Specifications Table

**Table:** 

Subject	Environmental Science, Oceanography, Molecular Biology
Specific subject area	Metabarcoding analyses of Foraminiferal biodiversity and spatial distribution
Data format	Raw data (fastq.gz file)
Type of data	Raw metabarcoding Data (fastq files) Analyzed Tables Figures
Data collection	In March 2023, surface sediment samples were collected using a 100 cc volume Van Veen Grab Sampler at variable depths. The DNA was extracted from each sediment sample using DNeasy Power Soil Kit (Qiagen) following manufacturer protocol. DNA fragments were amplified and sequenced using the Illumina HiSeqX platform.
Data source location	Country: India State: Goa Locations: Mandovi and Chapora estuary, Goa, India.
Data accessibility	Repository name: SRA of NCBI Data identification number: PRJNA1040471 Direct URL to data: https://www.ncbi.nlm.nih.gov/bioproject/PRJNA1040471

## 1 Value of the Data

• The generated environmental DNA (eDNA) dataset provides the first-ever insight into the uncharted genetic diversity of foraminifera from tropical locations along the west coast of India.
• This dataset may also serve as baseline information as a foraminiferal proxy for monitoring the long-term effect of mining pollution on the health of estuaries of the state of Goa, India.
• This dataset may serve as reference information for metabarcoding-based biomonitoring, biodiversity assessment surveys, and environmental impact assessment studies from other Indian marine habitats across time and space.
• The metabarcoding data hold the potential to aid predictive analysis, which is essential to understanding the effect of marine pollution on microorganisms.

## 2 Background

Goa, a state of the Republic of India, is among the primary producer of iron ore. The opencast iron ore mining at Goa creates a huge pile of leftover dumps and rejects, which enter into the estuaries during the Indian monsoon season. To minimize miningʼs harmful consequences before they reach humans, it is critical to assess them through the perspective of lower-level species in the food chain. Foraminifera are widespread marine meiofauna-sized protists widely used to evaluate environmental quality [4]. Owing to the short life cycle and responsiveness to environmental stresses, foraminifers have proven to be an effective proxy in biomonitoring marine environments [5]. The wet-picking method preserves the soft-bodied and/or finely agglutinated test of monothalamous foraminifera [1,2,6]. On the other hand, ecotoxicological studies use the dry-picking method, where only hard-shelled foraminifers were microscopically detected in marine sediments [7], which limits the benthic foraminifera to their full potential in biodiversity and pollution monitoring studies. Foraminiferal environmental DNA (eDNA) metabarcoding approaches based on high-throughput sequencing techniques can provide valuable insight into the uncharted diversity of foraminifers in ecologically sensitive marine environments and thus have considerable implications in biomonitoring surveys tracing anthropogenic impacts [8]. Here, we document the first insight into the uncharted diversity of organic-walled benthic foraminifera, which outnumbered the hard-shelled foraminifers in the sediment samples analyzed using metabarcoding approaches. To our understanding, no monothalamous foraminifera sequences have been reported from Goa, India. This work aimed to establish a metabarcoding dataset of foraminifera from the tropical location along the west coast of India that can be used as a reference for a wide range of eDNA-based studies from Indian marine habitats.

## 3 Data Description

**Fig. 1:**
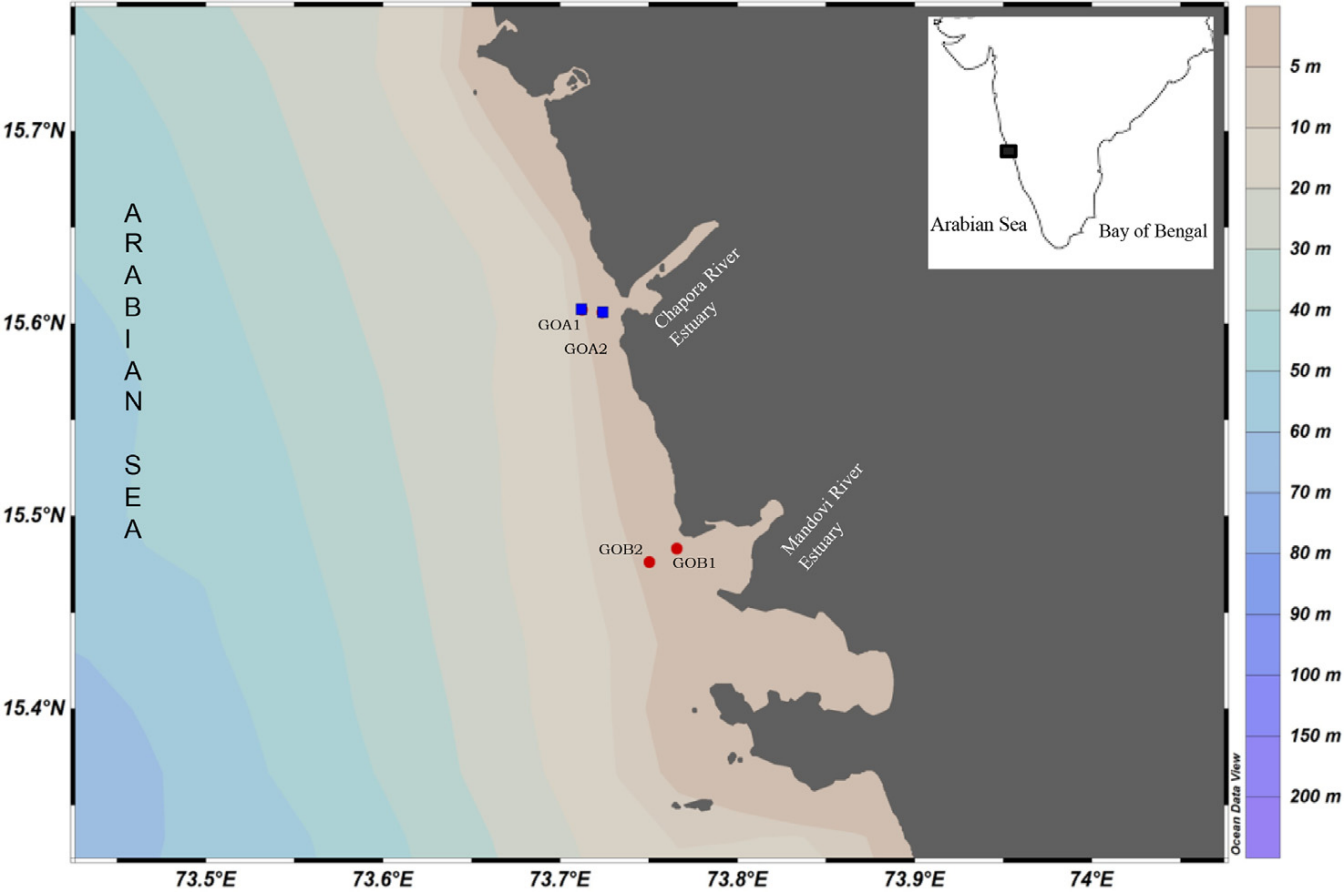
The study area and sampling stations.

The samples were collected during the premonsoon season (March 2023) from coastal regions of Goa, India (Fig. 1). The dataset described in this article is of foraminiferal genetic diversity including hard-shelled Globothalamea and soft-bodied monothalamids which were elucidated from the coastal shallow marine sediments using eDNA approach representing two mining affected estuaries of Goa, India, along the west coast of India. Besides, in-situ measurements of physical environmental parameters, including temperature, pH, DO, salinity, and sediment granulometry, have been deduced. The Chapora estuary stations (GOA1 and GOA2) are affected by anthropogenic activities, such as fishing, sand mining, and the flow of untreated effluents and solid waste. On the other hand, the Mandovi estuary stations (GOB1 and GOB2) are affected by severe metal pollution in the surficial sediments caused due to Fe—Mn ore mining activities at Goa, India [9]. The sampling station coordinates, sampling depths, and in-situ environmental parameters recorded during sampling can be found in Table 1.

**Table 1:** Geographical coordinates, depths, and in-situ environmental parameters of sampling stations.

Station	Sampling depth [meters]	Latitude [°N]	Longitude [°E]	pH	Salinity [‰]	Temperature [°C]	DO [mg l^−1^]	Sediment granulometry
GOA1	5.18	15°36′21.3″	73°43′24.8″	7.8	32.6	27.8	4.34	Silty clay
GOA2	5.48	15°36′26.6″	73°42′43.8″	7.9	32.6	27.8	4.61	Silty clay
GOB1	6.09	15°28′59.9″	73°45′56.9″	8.2	33.8	25.5	3.58	Silty sand
GOB2	5.79	15°28′34.0″	73°45′00.5″	8.2	33.8	25.5	3.64	Silty sand

**Fig. 2:**
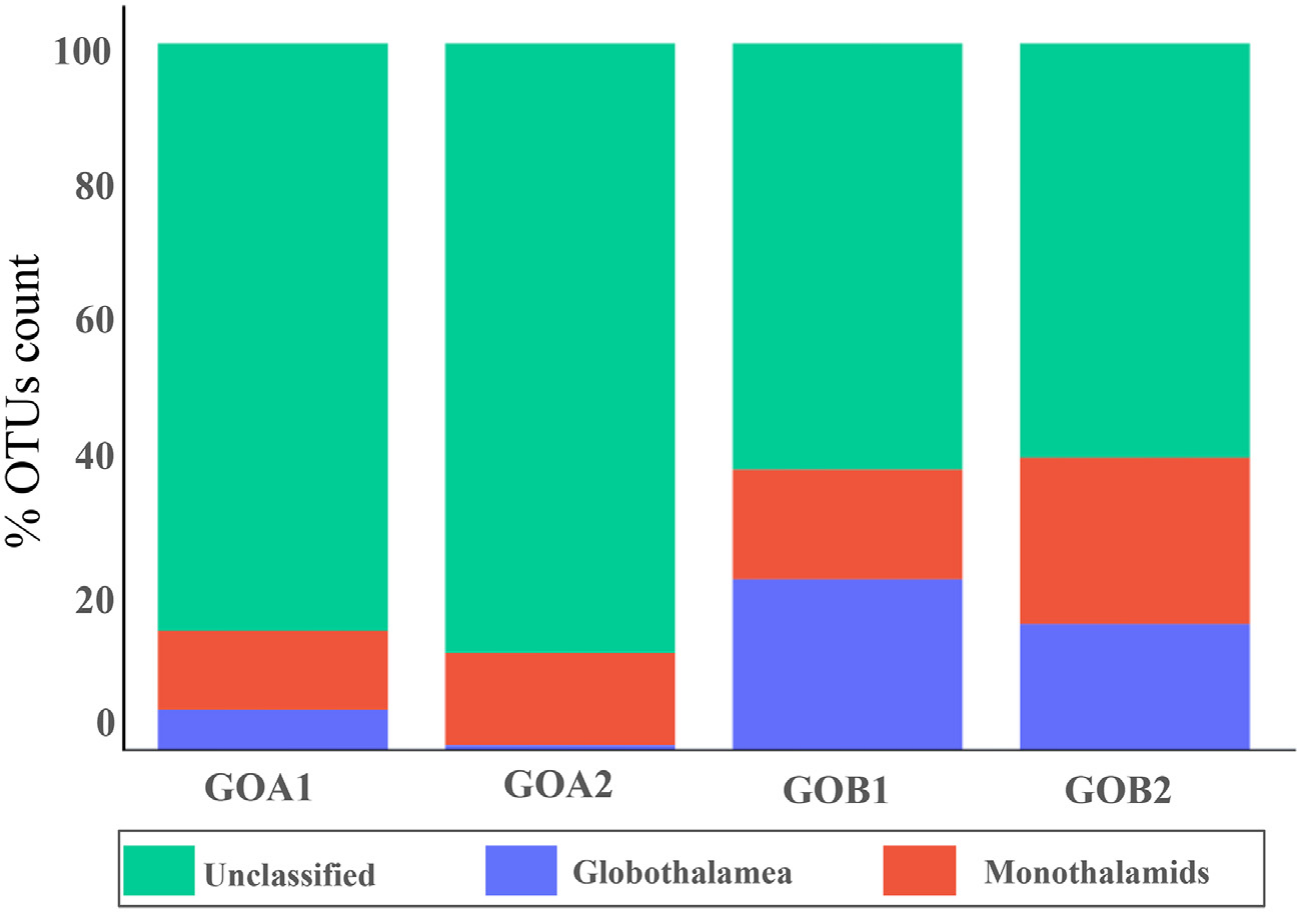
Relative abundance of OTUs at Class level, including unassigned OTUs (green) for all samples in the dataset.

**Fig. 3:**
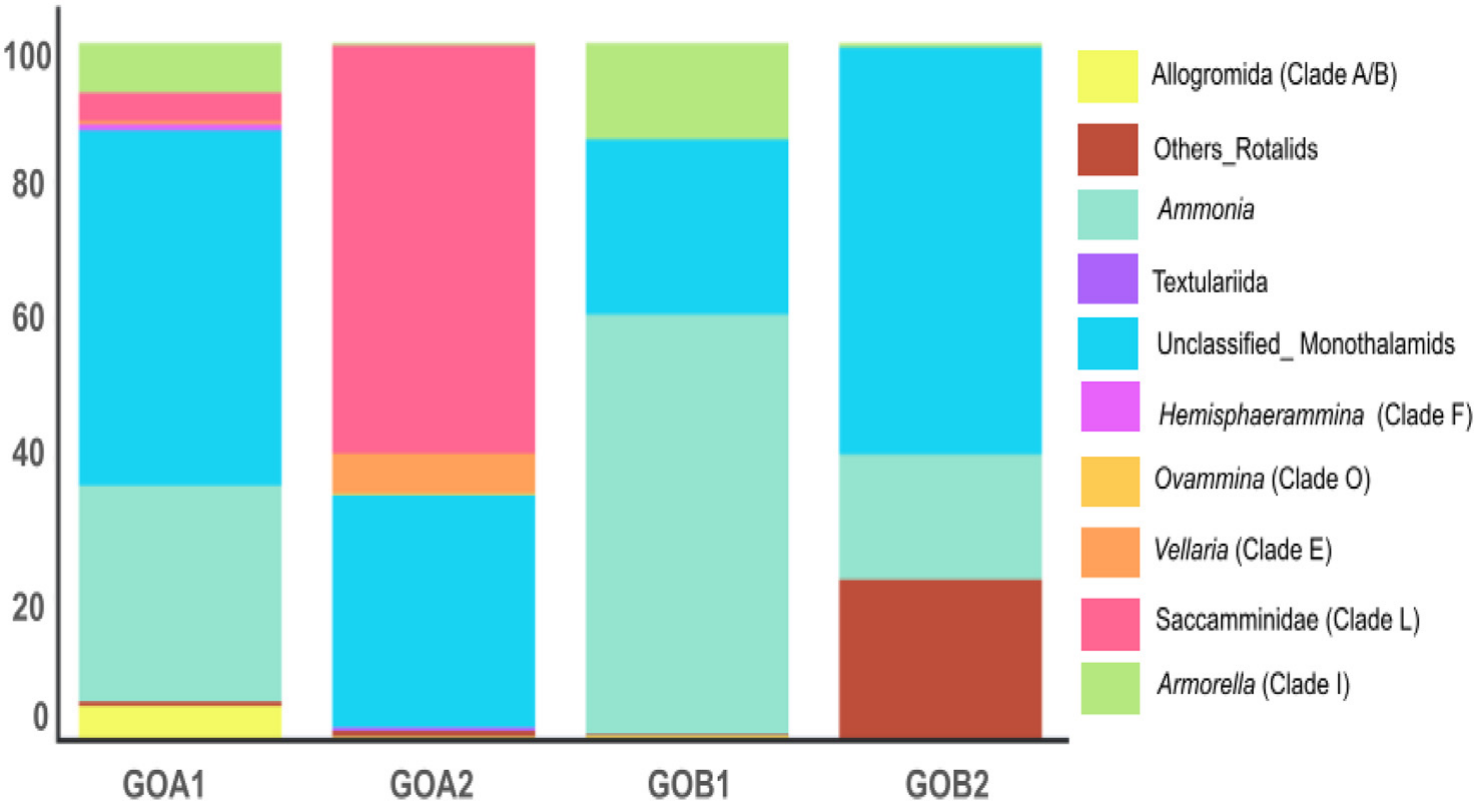
Relative abundance of OTUs at Order and Genus level, excluding unassigned OTUs for all samples in the dataset.

The dataset consists of raw environmental DNA (eDNA) reads from coastal regions of Goa, India. We sequenced the foraminifera-specific 37f region of 18S rRNA using the Illumina HiSeqX platform and classified the reads taxonomically using the QIIME2 pipeline [10]. The complete dataset numbered 6353,543 reads with 12,057 pair-end sequences with a length of nearly 200 bp. Of these, 10,893 were successfully taxonomically classified. De-novo clustering at 95 % resulted in 456 OTUs (Table 2). Our data suggests greater alpha diversity from Chapora estuary stations compared to Mandovi estuary stations. More unclassified foraminiferal sequences were documented from the Chapora estuary (57 %) than from the Mandovi estuary (19.67 %). The data without unclassified OTUs showed that in the Chapora estuary, GOA1 has higher OUTs counts for soft-bodied monothalamids (66 %) than multichambered globothalamids (33 %). At station GOA2, monothalamous has a maximum OTUs abundance of 95 % (Fig. 2). To the contrary, in Mandovi estuary, GOB1 station has maximum OTUs abundance for globothalamids, and at station GOB2, both the monothalamids and globothalamids OTUs abundance was comparable (Fig. 2; Table 3). The results of taxonomic classification at the genus level are presented in Fig. 3.

**Table 2:** Filtering statistics through the QIIME 2.0 pipeline.

Station Name	Input Reads	Filtered Reads	Denoised	Merged	Non-Chimeric	No of OTU reads (95 % clustering)	Unique OTUs
GOA1	1,437,554	1,414,407	1,392,931	2943	2740	5246	145
GOA2	1,974,791	1,924,297	1,893,785	5020	4560	8701	181
GOB1	1,427,742	1,391,052	1,384,440	2224	1916	3261	52
GOB2	1,513,456	1,468,118	1,446,107	1870	1677	3052	78

**Table 3:** Class, order, and genus level OTUs distribution across sampling locations.

Taxonomic level	GOA1	GOA2	GOB1	GOB2
Globothalamea	152	28	461	298
Others_Globothalamea	0	20	0	26
Others_Rotaliida	3	5	2	152
*Ammonia*	149	0	459	120
Textulariida	0	3	0	0
Monothalamids	305	592	297	395
Unclassified_Monothalamids	245	201	192	391
*Hemisphaerammina* (Clade F)	4	0	0	0
*Ovammina* (Clade O)	0	2	0	0
*Vellaria* (Clade E)	2	34	0	0
*Saccamminidae* (Clade L)	20	353	0	0
*Armorella* (Clade I)	34	2	105	4

## 4 Experimental Design, Materials and Methods

### 4.1 Sampling

Surface sediment samples were collected using the Van Veen Grab Sampler in March 2023. The upper 2 cm sediment layer has been sampled from the surface of approximately 10 cm^2^. Samples for foraminiferal environmental DNA (eDNA) were immediately transferred to sterile 15 ml falcon centrifuge tubes and frozen in a −20 °C deep freezer.

### 4.2 Foraminiferal environmental DNA (eDNA) analysis

The DNA was isolated from 250 mg of bulk sediment with DNeasy Powersoil Kit (Qiagen) in duplicates and later pooled to concentrate the DNA. Foraminiferal-specific 37f hypervariable region of 18S rRNA gene fragment was targeted and PCR amplified with the s14F1 (5′-XXXXXAAGGGCACCACAAGAACGC-3′) and s15r (5′-XXXXXCGGTCACGTTCGTTGC-3′) primers tagged with sequences of 5 nucleotides appended at their 5′ ends [[11], [12], [13]]. For each sample, PCR was performed in triplicates. Amplicons were quantified using a Qubit 3.0 fluorometer, and the pool was purified with a Flavogen PCR purification kit. Library preparation was performed with TruSeq DNA PCR-Free LT Library Prep Kit (Illumina) and was loaded onto a HiSeqX instrument for a paired-end HTS run.

### 4.3 Post-sequencing data processing

Raw sequence data were processed using the QIIME2 pipeline. Adapters and tags were removed using Cutadapt 2.0 [14]. Sequence reads were quality-checked and merged, and amplicon sequence variants (ASVs) were generated using the DADA2 plugin with -trunc-len-f 123 and -p-trunc-len-r 91 [15], as implemented in the QIIME 2.0 pipeline. ASVs were taxonomically classified using the PR2 database version 4.14.0 [16] and implemented in the Naïve Bayes classifier to annotate taxonomic information as OTUs. The results were presented as OTUs-to-sample tables.

## Limitations

Not applicable

## Ethics Statement

The authors have read and follow the ethical requirements for publication in Data in Brief and confirming that the current work does not involve human subjects, animal experiments, or any data collected from social media platforms.

## CRediT authorship contribution statement

**Tushar Kaushik:** Conceptualization, Methodology, Funding acquisition, Writing – original draft, Writing – review & editing. **Vaishnavi Dixit:** Data curation, Software, Writing – original draft, Writing – review & editing.

## Data Availability

Dataset of Foraminiferal sedimentary DNA from West coast of India (GOA), Nov 15 '23 (Original data) (NCBI).

## Appendix Supplementary materials

Image, application 1

Image, application 2
